# Novel 199 base pair *NEFH* promoter drives expression in retinal ganglion cells

**DOI:** 10.1038/s41598-020-73257-z

**Published:** 2020-10-05

**Authors:** Sophia Millington-Ward, Naomi Chadderton, Megan Berkeley, Laura K. Finnegan, Killian S. Hanlon, Matthew Carrigan, Peter Humphries, Paul F. Kenna, Arpad Palfi, G. Jane Farrar

**Affiliations:** 1 The School of Genetics and Microbiology, Trinity College Dublin, Dublin 2, Ireland; 2 The Research Foundation, Royal Victoria Eye and Ear Hospital, Dublin 2, Ireland

**Keywords:** Genetics, Molecular medicine

## Abstract

Retinal ganglion cells (RGCs) are known to be involved in several ocular disorders, including glaucoma and Leber hereditary optic neuropathy (LHON), and hence represent target cells for gene therapies directed towards these diseases. Restricting gene therapeutics to the target cell type in many situations may be preferable compared to ubiquitous transgene expression, stimulating researchers to identify RGC-specific promoters, particularly promoter sequences that may also be appropriate in size to fit readily into recombinant adeno associated viral (AAV) vectors, the vector of choice for many ocular gene therapies. In the current study we analysed EGFP expression driven by various sequences of the putative human *NEFH* promoter in order to define sequences required for preferential expression in RGCs. EGFP expression profiles from four different potential *NEFH* promoter constructs were compared in vivo in mice using retinal histology and mRNA expression analysis. Notably, two efficient promoter sequences, one comprising just 199 bp, are presented in the study.

## Introduction

Inherited ocular disorders have been at the forefront of the field of gene therapy; particularly given the recent market authorisation of Luxturna in the US and Europe, a recombinant adeno associated virus (AAV) 2 therapy for biallelic RPE65-linked inherited retinal degenerations^1^. To date there are approximately 30 clinical trials for recombinant AAV therapies for ocular indications (clinicaltrials.gov). Restricting expression of a gene therapy to the target tissue or target cell type, in principle, should provide significant potential safety benefit, as well as possibly increasing the efficacy of the therapy^2,3^. The use of well characterised AAV serotypes with specific cell tropisms, cell/tissue specific promoters as well as surgical procedures to deliver the therapy locally all represent ways of achieving this. For example, AAV2 is known to target retinal ganglion cells (RGCs) efficiently following intraocular injection^4–7^.

We previously demonstrated that a novel 2.2 kb murine Neurofilament heavy (*Nefh*) gene promoter preferentially and efficiently drove *EGFP* expression in RGCs and amacrine cells (ACs), compared to a ubiquitous *CMV* promoter^8^ when administered intravitreally in adult wild type mice using AAV2. Others too have investigated promoter sequences that express preferentially in RGCs. However, many promoter sequences of genes known to be RGC-specific, such as *Brn3a*, *Thy1* and *Rbpms*, are too large to be incorporated into AAV vectors, the vector of choice for many ocular gene therapies. AAV has a maximum cargo capacity of approximately 4.7 kb^8–13^. One study demonstrated that the *DCX* promoter (3310 bp; doublecortin) can drive strong and specific RGC expression following AAV-QuadYF-mediated delivery^14,15^. In addition, Simpson et al.^16^ analysed various promoters with similar expression profiles and found the *NEFL* (2693 bp; neurofilament, light polypeptide) promoter to be the most specific to RGC in mice and NHPs. Following intravitreal (IV) delivery, high levels of gene expression from a 1 kb upstream sequence of the SNCG gene was shown to co-localise with Brn3A indicating preferential expression in RGCs. However this promoter was not further refined^6^.

Clearly, when treating a human condition, employing a human DNA sequence, rather than the murine *Nefh* promoter sequence, may be preferable in terms of utilising the human transcription and translation machinery. Thus in the current study we analysed upstream sequences of the human Neurofilament Heavy (*NEFH*) gene to establish regions that convey efficient tissue specificity. The focus was also on identifying a smaller ‘RGC promoter’ that as part of a gene therapy construct would readily fit within the 4.7 kb confines of the AAV packaging capacity. Since AAV2 is the vector of choice for targeting rodent RGCs^17–19^ and in addition is known from Luxturna and various clinical trials to be well tolerated in the human eye (Ref.^1^, clinicaltrials.gov), this serotype was utilised in the current study.

## Results

**Figure 1:**
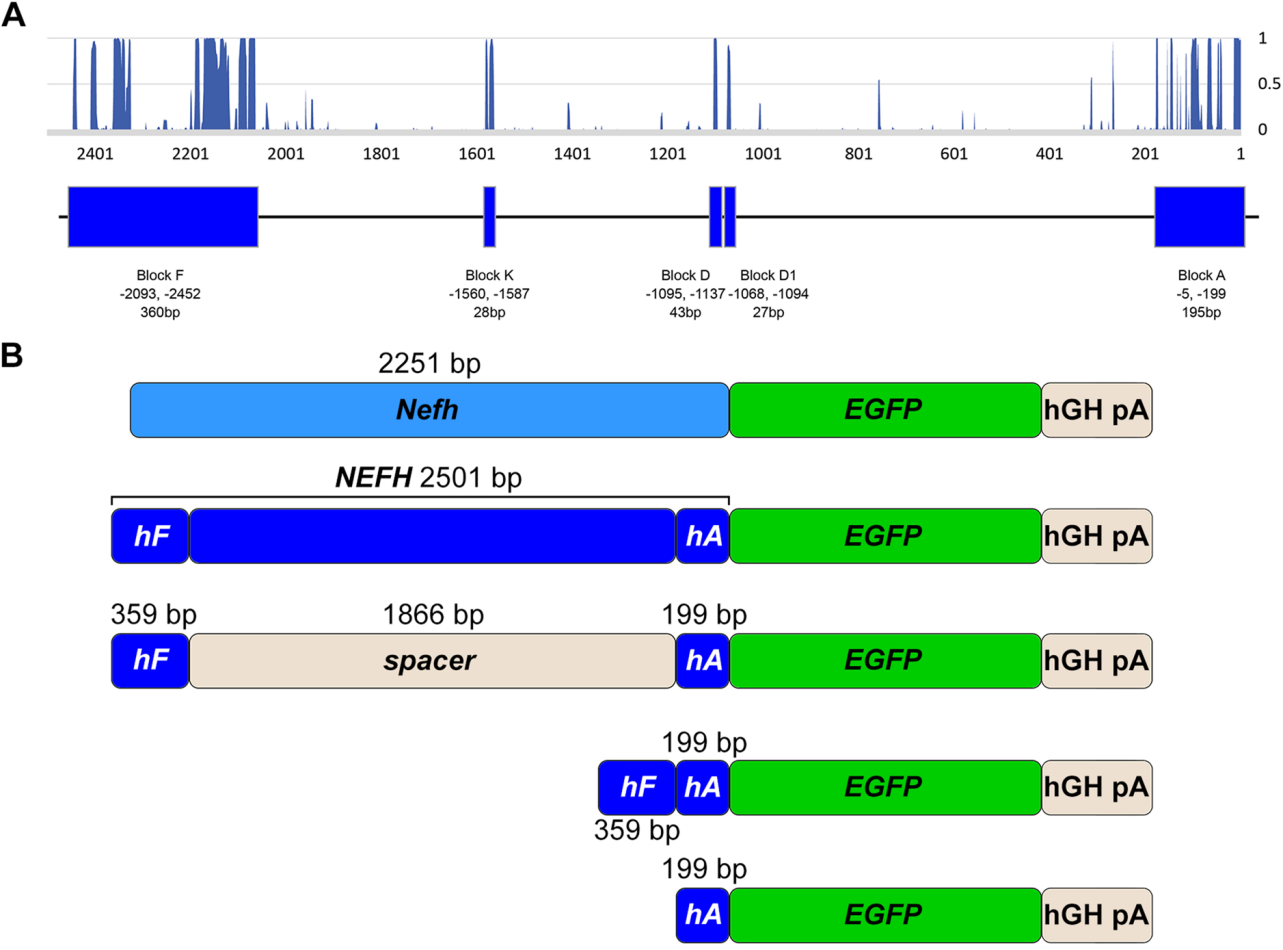
*NEFH* upstream sequence analysis and construct design. (**A**) Sequence conservation between 40 placental mammal species within the 2.5 kb region directly upstream of the human *NEFH* transcriptional start site, i.e. the putative human *NEFH* promoter region. Conservation level between 0 and 1 was assigned to each nucleotide. Units of conserved regions have been defined as Blocks F, K, D, D1 and (**A**,**B**) Schematic representation of human *NEFH* promoter constructs driving an *EGFP* cDNA. Conserved regions *hF* and *hA* consisting of Block F and Block A, and the spacer are indicated. The murine *Nefh* promoter is also given for reference. Promoter element sizes are given in base pairs (bp).

Significant advances have been made in the development of gene therapies for ocular disease; indeed the first in vivo AAV gene therapy to receive market authorisation was Luxturna for RPE65-linked recessive inherited retinal degenerations^1^. Clearly, using promoters that restrict transgene expression to the target cell or tissue type may represent advantages in terms of potential safety and efficacy in gene therapy approaches. Previous studies suggested that the murine *Nefh* mRNA is one of the most highly enriched transcripts in RGCs with an enrichment factor (EF) of 245-fold in the associated analysis^8,10^. We established that a 2.2 kb murine *Nefh* promoter (*Nefh-P*) driving an *EGFP* gene, not only provided high levels of transgene expression in the ganglion cell layer (GCL) but also enabled highly preferential expression in RGCs with only a minimal number of ACs expressing the EGFP transgene. The objective of the current study was the characterisation and in vivo evaluation of a human *NEFH* promoter for potential use in AAV-mediated gene therapies. In the current study a 2.5 kb region of upstream sequence of the human *NEFH* gene was compared between 40 placental mammalian species using an in silico pipeline, which was developed specifically to isolate basewise conservation data from UCSC. Conservation data ranged from 0 to 1 for a given base, where 0 represents no significant conservation between mammals and 1 indicates complete conservation. Results of this analysis were plotted to visualise conserved regions (Fig. 1A); the rational being that sequence conservation between mammalian species might indicate functionally important sequences. Employing this methodology, two highly conserved regions, termed Block F and Block A were identified. 2.5 kb of the *NEFH* upstream region, encompassing both Block F and Block A and the endogenous sequence in between were then cloned upstream of an *EGFP* cDNA (*NEFH-P*). Additionally, the Block F and Block A sequences were generated in a construct with a non-functional spacer sequence between the blocks and cloned upstream of the *EGFP* cDNA (*hF-sp-hA-P*). Notably this spacer sequence was designed to be the same length as the endogenous sequence. Blocks F and A were also engineered in one construct such that they were juxtaposed and upstream of the *EGFP* cDNA (*hF*-*hA-P*). Finally, Block A was cloned on its own upstream of the *EGFP* cDNA (*hA-P*; Fig. 1B). High titre preparations of AAV2/2 expressing *NEFH-P*, *hF*-sp-*hA-P*, *hF*-*hA-P* and *hA-P* were generated and *EGFP* expression profiles compared following subretinal (SR) or intravitreal (IV) injection into eyes of wild type mice. Full sequences of human *NEFH-P*, indicating conserved Block F and conserved Block A, and *hA-P* are provided in Supplemental Table 1.

### Histological analysis of promoter activity

**Figure 2:**
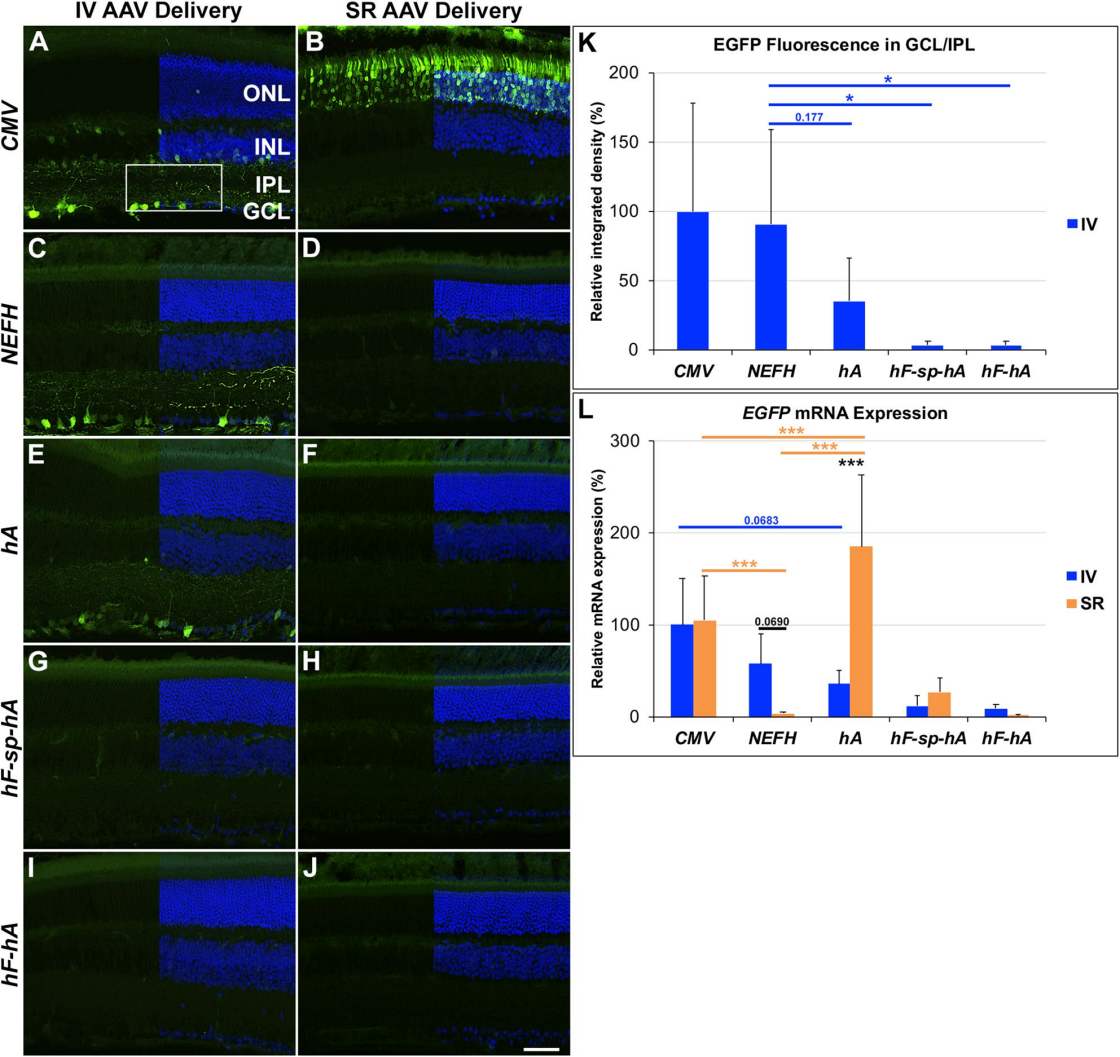
Native EGFP expression following intravitreal (IV) and subretinal (SR) injection of AAV delivered *NEFH* promoter constructs. 1 × 10^9^ vg of *CMV-P, NEFH*-*P*, *hA*, *hF*-*sp*-*hA* or *hF*-*hA* vectors were delivered by IV or SR injection to adult wild type mice and analysed 4 weeks post-injection. For histology, eyes were enucleated, fixed in 4% pfa, cryosectioned and stained with DAPI. Green and blue label correspond to native EGFP and DAPI fluorescence, respectively. Native EGFP fluorescence was detectable from *CMV-P* (**A**), *NEFH-P* (**C**) and *hA-P* (**E**) following IV injection. When delivering SR, native EGFP fluorescence was detectable from the *CMV-P* construct (**B**). *ONL* outer nuclear layer, *INL* inner nuclear layer, *IPL* inner plexiform layer, *GCL* ganglion cell layer. Scale bar (**J**) 50 μm. (**K**) Integrated fluorescence intensity of EGFP label was quantified in the GCL and IPL layers (as exemplified by the white rectangle in **A**) in the IV injected retinas (using cellSens); relative values are given as mean + SD in a bar chart (*CMV-P* was taken as 100%), n = 4–5, *p < 0.05 (*t*-test). (**L**) mRNA was purified from transduced whole retinas and EGFP expression determined using RT-qPCR; Actb was used as internal control. Relative *EGFP* mRNA expression levels from *CMV-P*, *NEFH*-*P*, *hA-P*, *hF*-*sp*-*hA-P* or *hF*-*hA-P* delivered IV (n = 11, 10, 12, 11, 5 respectively) were compared to expression levels following SR delivery (n = 10, 13, 10, 5 respectively) ; mean + SD [compared to *CMV-P* IV delivery (n = 5)] are given in a bar chart, n = 5–12, ***p < 0.001 (ANOVA); p values are not given for *hF-sp-hA-P* and *hF-hA-P*.

Following both IV and SR injection of various RGC promoter constructs, native EGFP-positive cells were assessed 4 weeks post AAV delivery. Native EGFP-positive cells were identified in the GCL and the inner plexiform layer (IPL), with *NEFH-P*, *hA-P* and *CMV-P* (Fig. 2A,C,E), but not with *hF-sp-hA-P* or *hF-hA-P* (Fig. 2G,I) when delivered IV, while photoreceptor cells were readily transduced with *CMV-P* when delivered SR and, to a lesser extent, EGFP-positive cells were found in the GCL, IPL and inner nuclear layer (IN; Fig. 2B). Notably, EGFP-positive cells were not detected with any of the *NEFH* promoter constructs following SR injection (Fig. 2D,F,H,J). Native EGFP fluorescence (normalised integrated fluorescence intensity) levels in the GCL and IPL following IV delivery of human *NEFH-P*, *hA-P*, *hF-sp-hA-P* and *hF-hA-P* promoter constructs were quantified and compared to EGFP fluorescence from *CMV-P* (100 ± 78.1%). Fluorescence levels were 90.9 ± 68.2%, 35.2 ± 30.8%, 3.4 ± 2.4% and 2.4 ± 2.4% respectively (Fig. 2K); Thus *NEFH-P* and *CMV-P* expressed at similar levels and the 199 bp *hA-P* provided approximately a third of *CMV-P* expression levels.

### *EGFP* mRNA analysis of promoter activity in murine retina

Initially, the human *NEFH-P* vector was compared to the previously published murine *Nefh-P* vector^8^. 1 × 10^9^ vg *NEFH-P* or *Nefh-P* were delivered by IV injection to eyes of wild type mice and *EGFP* mRNA expression compared 4 weeks post-injection using RT-qPCR in whole retinal RNA samples. The level of *EGFP* mRNA from human *NEFH-P* (39.3 ± 21.8%; n = 10), while still robust, was ~ 2.5-fold lower (p < 0.05) than from murine *Nefh-P*. Both promoter constructs were also injected SR into wild type mice and EGFP expression levels analysed by RT-qPCR 4 weeks post-injection. Levels of *EGFP* expression from both *NEFH-P* and *Nefh-P* were reduced significantly, by 93.4% and 92.5% respectively, when delivered SR compared to IV suggesting high level of specificity could be provided by the murine and human *Nefh* promoters (n = 10 and n = 5 respectively, p < 0.001).

Next, 1 × 10^9^ vg of *CMV-P*, human *NEFH-P*, *hF-sp-hA-P*, *hF-hA-P* and *hA-P* were delivered either IV or SR into adult wild type mice and compared to mRNA expression from *CMV-P* 4 weeks post-injection (Fig. 2L). Levels of *EGFP* mRNA expression from IV *CMV-P* were taken as 100% and all other expression levels are given relative to this. Interestingly, levels of *EGFP* expression from *CMV-P* were not significantly different when delivered IV or SR (100 ± 51.0%, n = 5 versus 105 ± 49.0%, n = 11). Expression from *NEFH-P* was somewhat lower than from *CMV-P* when delivered IV, but this was not significant (57.0 ± 32.1%, n = 10). However, when delivered SR *EGFP* expression from *NEFH-P* was reduced to 3.8 ± 1.2% (n = 10) indicating high levels of specificity (p < 0.001 when compared to *CMV-P* SR).

AAV constructs with *hA-P* and human *NEFH-P* expressed similar levels of *EGFP* when 1 × 10^9^ vg were delivered IV (35.6 ± 14.8% versus 57.0 ± 32.1% relative to *CMV-P*, n = 12), but expression from *hA-P* was somewhat less than from *CMV-P* IV (p < 0.05). In contrast, when delivered SR, *hA-P* expressed *EGFP* at significantly higher levels, i.e. 185.0 ± 77.8% than *CMV-P* delivered SR (n = 13, p < 0.05). Expression levels from *hF-sp-hA-P* and *hF-hA-P* IV were significantly lower at 12.3 ± 11.5%, n = 11 and 8.99.0 ± 4.82%, n = 5, respectively, than from *CMV-P* (p < 0.05). These constructs were also significantly lower than *NEFH-P* (p < 0.01) and *hA-P* (p < 0.001). After SR injection, these two constructs expressed 27.3 ± 14.6% (n = 5) and 1.63 ± 0.72% (n = 5), respectively, compared to *CMV-P*.

### Immunocytochemistry analysis of promoter activity

**Figure 3:**
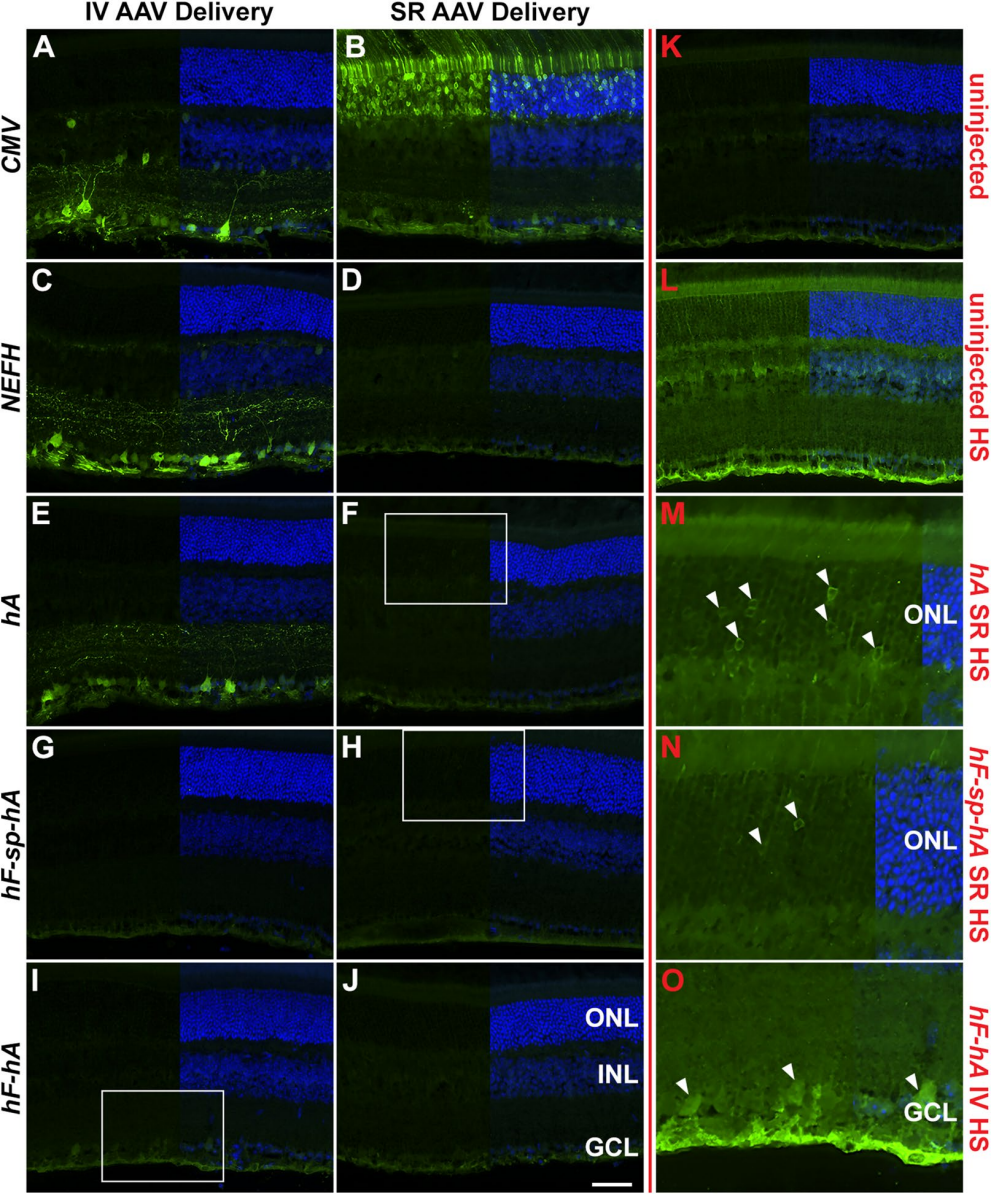
EGFP expression following intravitreal (IV) and subretinal (SR) injection of AAV delivered *NEFH* promoter constructs utilising EGFP immunocytochemistry. 1 × 10^9^ vg of *CMV-P, NEFH*-*P*, *hA-P*, *hF*-*sp*-*hA-P* or *hF*-*hA-P* vectors were delivered by IV or SR injection to adult wild type mice and analysed 4 weeks post-injection. For histology, eyes were enucleated, fixed in 4% pfa and cryosectioned. EGFP immunocytochemistry was performed utilising chicken anti-EGFP primary (Abcam), Alexa-Fluor-488 conjugated secondary antibodies (green) and DAPI nuclear stain (blue). Strong label was detected with IV delivery for *CMV-P* (**A**), *NEFH-P* (**C**) and *hA-P* (**E**) and with SR delivery in *CMV-P* (**B**) samples; there was no label in the uninjected retinas (**K**). At higher sensitivity levels [HS in labels; (**L**–**O**)], minimal transduction of photoreceptor cells with SR delivery of *hA-P* (**M**) and *hF-sp-hA-P* (**N**) and retinal ganglion cell with IV delivery of *hF-hA-P* (**O**) constructs was detected (white arrowheads; white rectangles in F, H and I indicate the areas depicted at HS in (**M**), (**N**) and (**O**), respectively); note the high background in the HS images as demonstrated by the uninjected retina (**L**). *ONL* outer nuclear layer, *INL* inner nuclear layer, *GCL* ganglion cell layer. Scale bar (**J**) 50 μm.

Distribution of native EGFP-positive cells in the retinal layers and mRNA expression analysis indicated that *CMV-P*, *NEFH-P* and *hA-P* were the most effective RGC promoters (Fig. 2). However, since high levels of *EGFP* mRNA were observed following SR injection of *hA-P*, EGFP protein expression from all promoter constructs was evaluated in more detail following both SR and IV delivery of 1 × 10^9^ vg of AAV constructs, using EGFP immunocytochemistry. No EGFP expression was observed from *hF-sp-hA-P* following IV injection (Fig. 3G). Similar expression profiles were observed, with strong EGFP label detected in the GCL layer, from *CMV-P*, *NEFH-P* and *hA-P* following IV (Fig. 3A,C,E respectively). A small amount of expression from *hF-hA-P* was observed following IV delivery (Fig. 3I). Only *CMV-P* expressed high levels of EGFP in the outer nuclear (ONL) layer following SR delivery (Fig. 3B). Images were also analysed at high sensitivity (HS) conditions (with increased exposure) in order to identify cells with low EGFP expression. Minimal EGFP expression could be observed in the ONLs of retinas that had received *hA-P* and *hF-sp-hA-P* via SR delivery (Fig. 3M,F,H,N, respectively). HS analysis of images also identified minimal RGC expression from *hF-hA-P* following IV delivery (Fig. 3O). No expression was determined from *NEFH-P* or *hF-hA-P* following SR injection with HS analysis indicating that expression from these promoters is restricted (Fig. 3D,J). No label was detected in uninjected retinas at either standard or HS analysis (Fig. 3K,L). Thus, like *CMV-P*, *NEFH-P* and *hA-P* expressed high levels of EGFP in the GCL following IV injection (Fig. 3A,C,E), but in contrast to *CMV-P,* EGFP expression in the ONL was minimal following SR delivery (Fig. 3B,D,F).

**Figure 4:**
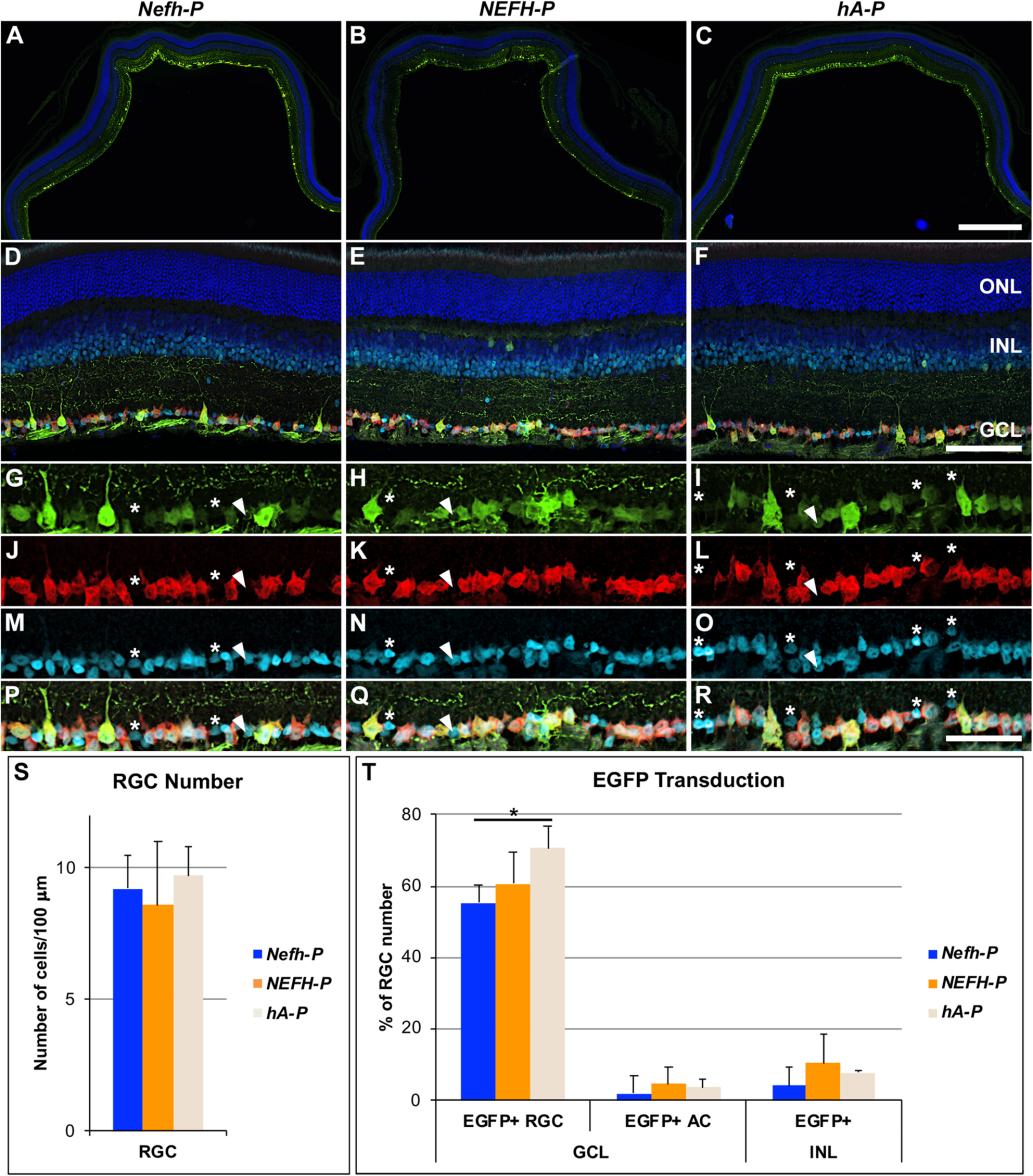
EGFP expression following IV delivery of *Nefh-P*, *NEFH-P* or *hA-P*. *Nefh-P, NEFH-P* or *hA-P* was delivered via IV injection (1 × 10^9^) to adult wild type mice (n = 4). Four weeks post-delivery, eyes were enucleated, fixed in 4% pfa and cryosectioned. Immunocytochemistry for EGFP (Alexa-Fluor-488 label, green), RBPMS (Cy3 label, red) and PAX6 (Cy5 label, light blue) was carried out. (**A**–**C**) Overview of EGFP-transduced retinas. (**D**–**F**) Overview of EGFP-, RBPMS- and PAX6-label in EGFP-transduced retinas. Distribution of EGFP (**G**–**I**), RBPMS (**J**–**L**), PAX6 (**M**–**O**) and their overlay (**P**–**R**) in the GCL is presented. Most cells were positive for the three markers demonstrating that they were EGFP-transduced RGCs (**G**–**R**). Arrowheads indicate amacrine cells (ACs, RBPMS negative/PAX6 positive) that expressed EGFP, while asterisk indicate ACs (RBPMS negative/PAX6 positive), which did not express EGFP. Bar chart representation of the number of RGCs in the transduced retinas (**S**) and the percentage of EGFP-positive RGCs and ACs in the GCL and the EGFP-positive cells in the INL (**T**); the number of RGCs was taken as 100%; bars represent mean + SD; *p < 0.05 (ANOVA). Automated (for RGC and INL cells) and manual (for ACs) quantification was carried out in cellSens. *ONL* outer nuclear layer, *INL* inner nuclear layer, *GCL* ganglion cell layer. Scale bars: 500 μm (**C**), 100 μm (**F**), 50 μm (**R**).

As the human *NEFH-P* and *hA-P* promoters were both efficient and highly preferential to RGCs we further analysed expression from these promoters in subsequent experiments. Specificity and efficacy of RGC targeting by *NEFH-P* and *hA-P* was determined following IV injection of 1 × 10^9^ vg of *NEFH-P* and *hA-P* AAV vectors in wild type retinas (Fig. 4). The previously described murine *Nefh-P* vector^8^ was used as a control. Transduced sections from the central retina were stained using immunocytochemistry for EGFP, RBPMS and PAX6 (Fig. 4A–R) and positive cells quantified. The number of RGCs detected were similar between the analysed groups (Fig. 4S), i.e. 9.2 ± 1.3, 8.6 ± 2.4 and 9.7 ± 1.1, per 100 μm, for murine *Nefh-P,* human *NEFH-P* and *hA-P* transduced retinas, respectively (n = 4).The percentage of EGFP-positive RGCs (RBPMS positive/PAX6 positive) was 55.0 ± 7.8%, 61 ± 9% and 71 ± 6.0% for *Nefh-P, NEFH-P* and *hA-P*, respectively (n = 4, Fig. 4T). While *NEFH-P* and *hA-P* did not differ statistically significantly, a significantly greater number of EGFP-positive RGCs was observed with *hA-P* compared to *Nefh-P* (*t*-test). The percentage of EGFP-positive ACs in the GCL (RBPMS negative/PAX6 positive, Fig. 4T) was much lower compared to EGFP positive RGCs and did not differ significantly between the three AAV promoter vectors; 2.0 ± 1.0%, 5.0 ± 4.6% and 4.0 ± 2.2% for murine *Nefh-P,* human *NEFH-P* and *hA-P*, respectively (n = 4). The percentage of EGFP-positive cells in the INL was also much lower compared to EGFP positive RGCs; 4.2 ± 2.7%, 10.5 ± 8.2% and 7.8 ± 0.70%, respectively (n = 4, Fig. 4T) and did not differ statistically significantly. In the above results, the number of RGCs in the GCL was set as 100% and all other numbers are given relative to this. Cells were quantified automatically in cellSens, except for ACs, which were counted manually.

**Figure 5:**
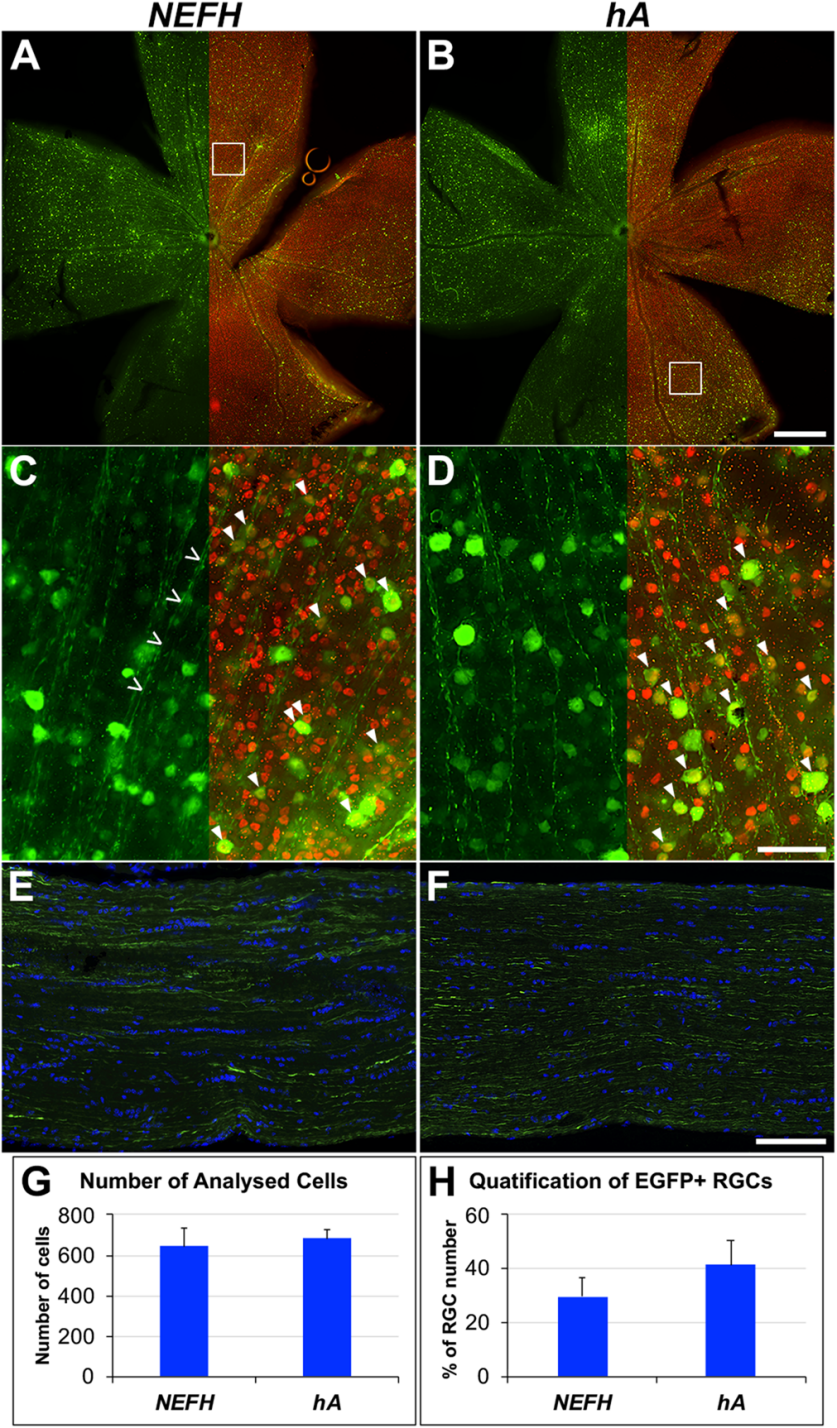
EGFP expression from *NEFH* promoter constructs in retinal wholemounts and optic nerves. 1 × 10^9^ vg of *NEFH-P* or *hA-P* AAV were delivered by IV injection to adult wild type mice, and EGFP positive cells analysed 4 weeks post-injection. Eyes were enucleated and fixed in 4% pfa; retinas wholemounted and optic nerves (ONs) cryosectioned longitudinally. Retinas were immunostained for BRN3A (Cy3 label, red) and EGFP (Alexa-Fluor-488 label, green). ONs were immunostained for EGFP (Alexa-Fluor-488 label, green), and counterstained with DAPI. (**A**,**B**) Overview of wholemount retinas with EGFP signal and BRN3A label (retinal ganglion cell, RGC) overlaid on the right half of the images. (**C**,**D**) Magnified view of wholemount retinas with EGFP signal and BRN3A label overlaid on the right half of the images. Some BRN3A-positive cells (RGCs) were co-labelled with EGFP; closed arrowheads indicate some double positive cells. Open arrowheads delineate an example of an EGFP-positive RGC axon. (**E**,**F**) EGFP-positive RGC axons detected in ONs. Scale bars: 500 μm (**B**), 50 μm (**D**), 100 μm (**F**). EGFP-positive RGCs were counted in six representative areas imaged at ×20 magnification (such as **C** and **D**) in each wholemount transduced with either *NEFH-P* or *hA-P* AAV (n = 6) using automated quantification in cellSens. The number of RGCs in the analysed areas (**G**) and the percent of EGFP-positive RGCs in the transduced retinas (**F**) are given in bar charts; bars represent mean + SD. Statistical significance between *NEFH-P* and *hA-P* AAV-transduced retinas was carried out using a *t*-test.

EGFP expression in RGCs was also analysed quantitatively in retinal wholemounts using the human constructs. *NEFH-P* and *hA-P* (1 × 10^9^ vg) AAV vectors were IV injected into adult wild type mice and retinal wholemounts (Fig. 5A–D) and optic nerves (ONs; Fig. 5E,F) assessed 4 weeks post-delivery using immunocytochemistry for EGFP and BRN3A (RGC). A significant proportion of the retinas were highly transduced; EGFP-positive RGCs were quantified automatically in 6 transduced areas of each wholemount using cellSens. The percentage of EGFP-positive RGCs following IV delivery of *NEFH-P* and *hA-P* promoter constructs was 29.7 ± 7.1% and 41.5 ± 9.2%, respectively, but this difference was not significant (n = 6; Fig. 5G,H). Note that the number of analysed RGCs/area was similar between the two groups, 632.7 ± 94.0 and 682.7 ± 45.3, respectively (Fig. 5G). Some axons from RGCs were also positive for EGFP in both the wholemounts and ONs (Fig. 5C,D,E,F). The percent of EGFP-positive RGCs determined in the wholemounts had a similar trend but lower numbers compared to those determined in the retinal sections; note that the same AAV dosage was used in both experiments. A likely explanation for this is that the wholemounts have significantly higher EGFP background levels than 12 μm thick retinal sections, hampering detection of low EGFP signal intensity cells. Therefore the efficacy of EGFP detection is higher in sections than wholemounts, resulting in higher numbers of EGFP-positive cells. (Note that detection efficacy of RGCs either with RBPMS or BRN3A is likely to be close to 100% in both wholemounts and sections as the signal/noise ratio for these labels is much higher.

## Discussion

A key objective of the study was to generate a valuable human RGC promoter, which provides preferential transgene expression in RGCs, but is minimal in size, thereby optimising its utility in gene therapy vectors such as AAV. Of note, Kim et al., 2006 analysed gene expression in RGC and non-RGC cells from post-mortem human retinas. They showed the greatest (~ 245-fold) enrichment of *NEFH* mRNA in RGC^8,10^ followed closely by *NEFL* (~ 150-fold enrichment) and *NEFM*.

Given the potential utility of such a promoter for future human gene therapies targeted to RGCs the focus of the current study was to analyse the human *NEFH* gene upstream sequence. This analysis resulted in the identification of two conserved regions, blocks A and F, which were subsequently included in various configurations in AAV vectors to drive *EGFP* reporter gene expression (Fig. 1). Because the *Thy1* promoter, which is known to drive RGC-specific expression, is only effective when a large ~ 6 kb spacer is included between the core promoter and enhancer elements^11,12^, we decided to include a spacer sequence between conserved regions F and A in one promoter construct. The spacer used was approximately the same length as the endogenous promoter sequence between conserved regions F and A.

In the current study we determined that the level of *EGFP* mRNA from the human *NEFH-P*, akin to the murine promoter, provided effective gene expression in murine RGCs. The data suggest that expression levels from the human *NEFH-P* both at the mRNA and protein level are in the same order of magnitude as levels provided by the *CMV* promoter (Fig. 2A,B,C,D,K,L) and similar to that seen for murine *Nefh-P*^8^. Compared to *CMV-P*, *NEFH-P* also provided high levels (57.8 ± 32.1%) of reporter gene expression in the GCL. Expression in the INL was minimal following IV injection into the murine eye. In contrast, levels of expression following SR injection of the AAV2/2 *NEFH-P* vector were barely detectable at the mRNA level (3.83 ± 1.24%) and were undetectable using histological analysis, highlighting the specificity of the *NEFH-P* for RGCs. Interestingly, the highly truncated *NEFH* promoter in the AAV2/2 *hA-P* vector (199 bp promoter) also enabled significant expression of *EGFP* following IV injection when evaluated by mRNA analysis (35.6 ± 14.8% compared to *CMV-P* IV), and the percentage of native EGFP positive cells in the GCL was 71.3 ± 3.13% compared to *CMV-P* IV. The 199 bp *hA-P* promoter was compared to the murine and human genome using BLAST. However, no more than 20% sequence identity was seen between it and other sequences, indicating that *hA-P* is unique to *NEFH*. It is of note that relatively high levels of *EGFP* mRNA expression were detected following SR injection of *hA-P*, yet only an insignificant amount of EGFP was found using EGFP immunocytochemistry (Fig. 3). This seems to indicate that the 199 bp *hA-P* promoter, while transcribed at high levels in the ONL, is not translated efficiently in these cells. Hence this very small promoter appears to convey RGC specificity by unknown post-transcriptional regulation.

Notably, a large proportion of cells in the transduced area of the GCL were EGFP-positive following IV injection of *NEFH-P* and *hA-P* (~ 50–70%, Fig. 4T). Only 2–5% of EGFP positive cells were ACs (EGFP positive/RBPMS negative/PAX6 positive; Fig. 4T). In addition, 5–10% of cells in the INL expressed EGFP. The identity of the EGFP-positive cells in the INL was not investigated but at least some of them appear to be ACs.

The preferential RGC expression exhibited by the promoter constructs described herein represent significant refinements for RGC gene therapies. Some promoters known to express specifically in RGCs have either not been expressed highly or are too large to be included in AAV vectors, which have a maximum cargo capacity of approximately 4.7 kb, e.g. *Brn3a*, *Thy1* and *Rbpms*^8–13^. Notably, the size of both the human *NEFH* promoter (2.5 kb) and, particularly, the truncated *hA-P* promoter (199 bp) are ideal for application within AAV vectors and thereby represents a significant advantage over the larger RGC promoters published to date^6,14–16^.

AAV2 incorporating the human connexin 36 (*CX36/GJD2*) promoter (2.8 kb) transduced NHP foveal ganglion cells. However, when shortened to 1.8 kb, expression was only seen in damaged retinas^5^. Jüttner et al.^20^ identified several synthetic promoters that target NHP or human post-mortem RGCs specifically, however, transduction varied between 1 and 13% of the total RGC population following SR injection and utilising AAV2/8BP2. It would be of interest to see whether the transduction efficacy could be improved with IV delivery. Interestingly, the authors found that less than 1% of the 230 synthetic promoter sequences tested by SR in mice replicated the specificity of their source genes^20^. The study also clearly showed how expression profiles from many of the same promoters varied between species (mouse, NHP and human retina). In the current study we have demonstrated that the RGC specificity of both the murine *Nefh*^8^ and human *NEFH* promoters was similar in mice. Simpson et al.^16^ described a 2693 bp *NEFL* promoter, which appears to have a similar expression profile to *NEFH-P* and *hA-P*, though notably *hA-P* is ~ 85% smaller than the *NEFL* promoter. It will however be important to evaluate *hA-P* and *NEFH-P* in NHP or human retina to evaluate whether the specificity of *hA-P* and *NEFH-P* obtained following IV delivery in mouse, is reflected in higher species; particularly as some promoters which express in the GCL in mice have been shown only to express in damaged NHP retinas, for example, the 0.48 kb hSYN and the 1.8 kb XC36/GJD2 promoters^5^.

AAVs have been utilised extensively for ocular gene delivery to animal models and in human clinical trials as well as for two in vivo licensed medicinal products; Luxturna and Zolgensma. For example, AAV2 and AAV9 are known to transduced RGCs in the perifoveal ring and to a lesser extent the peripheral retina in NHPs^5,16^ and clinical trials for LHON have demonstrated that AAV2 transduces RGCs efficiently in humans and indeed provides beneficial effects^7,21^. Additionally, a plethora of newer AAV serotypes have been shown to infect primate or human RGCs with greater efficacy, demonstrating enhanced tropism for both the foveal centre and peripheral retina. These include AAV2BP8^22^, Anc80^23^, AAV2/8 Y733F^24^, AAV2/2 quadYF^25,26^ and AAV7m8^22,26,27^, among others.

RGC delivery may be enhanced through incorporation of novel peptide insertions into VP1. Modification of AAV2 VP1 with peptides (NNPTPSR or GLSPPTR), which are applicable to any serotype, resulted in greater EGFP expression than AAV7m8^28^. Offering the potential for insertion of such peptides into VP1 of the most efficient RGC transducing serotypes to further boost expression. These novel serotypes, or a combination thereof, may provide the enhanced transduction required to effectively treat RGC-based disorders such as glaucoma, DOA, LHON, among others (reviewed in Ref.^29^), and may provide the breadth of delivery needed for the effective utilisation of novel technologies such as optogenetics, for example (reviewed in Ref.^30^). In such cases, the ability to restrict transgene expression to RGCs while still providing effective transgene expression, as demonstrated herein, would be highly advantageous to minimise potential off-target effects and could thereby improve safety and efficacy.

The current data suggest that *NEFH-P* drives highly specific expression in the RGC layer, with minimal expression observed in the INL or ONL, even following SR injection. In addition, when *NEFH-P* was reduced by over 90% (*hA-P*), the expression profile remained substantially the same. The 199 bp *hA-P* promoter therefore represents a very interesting and potentially versatile promoter that easily fits within the confines of the 4.7 kb transgene capacity of AAV vectors, thereby enabling delivery of larger genes while still providing efficient and highly preferential transgene expression in RGCs following IV injection.

## Materials and methods

### In silico analysis of the human *NEFH* promoter

To define a putative human *NEFH* promoter region, data from the University of California Santa Cruz (UCSC) genome browser (mm10 mouse mammalian conservation track^31^) were used to establish the conservation upstream of the transcriptional start site of *NEFH* gene. An in silico pipeline^8^, was used to isolate basewise conservation data (conservation data ranged from 0 to 1 for a given base, where 0 represents no significant conservation between mammals and 1 indicates complete conservation). Sequences from 40 placental mammal species (Supplementary Table S1, Ref.^8^) were used for conservation alignment and a graph plotted to visualise conserved regions of the human *NEFH* upstream sequence (Fig. 1A).

### Constructs and viral production

A murine 2.2 kb *Nefh* promoter driving *EGFP* expression (*Nefh-P*) was generated as described previously^8^. To generate a human *NEFH* promoter equivalent a 1.9 kb fragment of human genomic DNA (was amplified using the following primer pair: 5′ AGATCATCTTAAGACGCGTTGCTGTCAGCTGCTTGTGA 3′ and 5′GAGGTACAGTGTTCTCCTAAC 3′). The purified PCR product was cloned into pcDNA3.1 + (Invitrogen); an additional fragment of custom synthesised DNA (GeneWiz, South Plainfield, USA), corresponding to bp − 5 to − 637 of the *NEFH* sequence, was cloned into the above 1.9 kb partial promoter to generated a full 2501 bp of contiguous *NEFH* upstream sequence (− 5 to − 2452 of NM_ 021076.3). The full 2501 bp *NEFH*, upstream sequence was then excised and cloned in place of the *CMV* promoter in pAAV-*CMV*-*EGFP*^2^ to create *NEFH-P*. To create *hA-P* (− 5 to − 199 of NM_ 021076.3), conserved region A was amplified from human genomic DNA using the following primer pair: 5′-ATCGATGACGCGTCTCTGACGCAGCGTCGATT-3′ and 5′-AGATCATGATATCGGCCTGAGCAGGTGCGCGA-3′ and the amplification product cloned in place of the *CMV* promoter in pAAV-*CMV*-*EGFP*^2^. To generate *hF-hA-P *(− 5 to − 199 and − 2093 to − 2452 of NM_021076.3), the sequence of the conserved regions was custom synthesised (GeneWiz, South Plainfield, USA) and cloned into pAAV.*CMV*-*EGFP*^2^ in place of the *CMV* promoter. *hF-sp-hA-P* (− 5 to − 199, 1866 bp of lambda, and − 2093 to − 2452 of NM_021076.3) was generated by amplifying 1866 bp of lambda DNA using the following primer pair (5′-ATCGATGTTTAAACTACTACCGATTCCGCCTAGT-3′ and 5′-ATGCATGTTTAAACAGGCATTTATACTCCGCTGG-3′) and cloning this amplification product between the conserved Blocks A and F in *hF-hA-P*. All *EGFP* genes also included an hGH poly A. Nucleotides included in constructs are indicated in Fig. 1 and Supplemental Table 1. Sanger sequencing was used to verify all plasmid constructs. For each candidate *NEFH* promoter construct, recombinant AAV2 viruses were generated. Genomic titres were determined, as described previously^32,33^.

### Intravitreal and subretinal injection of mice

Wild type 129 S2/SvHsd mice (Harlan UK Ltd, Oxfordshire UK) were maintained under specific pathogen free conditions. Injections were performed in strict compliance with the European Union (Protection of Animals used for Scientific Purposes) Regulations 2012 (S.I. no. 543 of 2012) and the Association for Research in Vision and Ophthalmology (ARVO) statement for the use of animals. Intravitreal injections were performed as described^8,34^ and subretinal injections were performed as described in Ref.^33^. 1.0 × 10^9^ vg of virus was injected into eyes and RNA and histological analysis performed 4 weeks post-injection.

### RNA analysis

Retinas were harvested 4 weeks post-injection and total RNA extracted as described^35–37^. In vivo expression levels of *EGFP* were determined by reverse transcription PCR (RT-qPCR) on a StepOne Real Time PCR System (Applied Biosystems, Foster City, CA, USA) using a one step QuantiTect SYBR Green RT-qPCR kit (Qiagen Ltd., Crawley, UK) and the following primer pair: 5′ TTCAAGAGGACGGCAACATCC 3′ and 5′ CACCTTGATGCCGTTCTTTCGC 3′. RT-qPCRs were performed twice in triplicate. Expression levels were normalised using the internal housekeeping gene β-actin (*Actb)* and the following primer pair: 5′ AGAGCAAGAGAGGCATCC 3′ and 5′ TCATTGTAGAAGGTGTGGTGC 3′. Standard curves of *Actb* were generated by serially diluting retinal RNA 5×. Standard curves of *EGFP* were generated by serially diluting plasmid DNA containing an *EGFP* gene 10×. A minimum of 4 points were used in all standard curves.

### Immunohistochemistry and microscopy

Mice were sacrificed, eyes enucleated and optic nerves collected. Tissue samples were fixed in 4% paraformaldehyde in PBS overnight then washed in PBS. Some of the retinas and the optic nerves were cryoprotected in 10%, 20% and 30% sucrose in PBS, embedded in OCT (VWR), cryosectioned (12 μm) and thaw-mounted onto Polysine slides (Thermo Scientific). For wholemounting, fixed and PBS-washed retinas were removed from the eyecups and processed for immunocytochemistry immediately. Immunocytochemistry was performed as described before^38^. Sections/wholemounts were incubated with primary antibodies for EGFP (ab13970, Abcam; 1:1000 dilution), RBPMS (abn1376, Millipore; 1:400 dilution), BRN3A (sc-8429, Santa Cruz; 1:200) and CHX10 (sc-374151, Santa Cruz; 1:200 dilution) overnight for sections and for 3 days for wholemounts at 4 °C. Sections/wholemounts were then washed in PBS incubated with secondary antibodies conjugated with Alexa-Fluor-488, Cy3 and Alexa-Fluor-647 (Jackson ImmunoResearch Laboratories) in 1:400 dilution for 2 h for sections and 2 days for wholemounts and nuclei counterstained with DAPI. Samples were covered using Hydromount (National Diagnostics).

Fluorescent microscopy was carried out utilising an Olympus IX83 inverted motorised microscope (cellSens v1.9 software, Olympus) equipped with a SpectraX LED light source (Lumencor) and an Orca-Flash4.0 LT PLUS/sCMOS camera (Hamamatsu). Samples were imaged using a 10 × plan fluorite and a 20 × plan super apochromat objective utilising enhanced focal imaging (EFI) with typically 5–8 Z-slices (30 slices in 20 × images for colocalisation in wholemounts) using cellSens. Lateral frames were stitched together for pan-retinal sections and wholemounts in cellSens.

Representative sections from each eye were taken from the central retina (within 250 μm of the optic nerve) and analysis performed in the central part of the stitched sections utilising cellSens; same settings were applied to all samples. Integrated fluorescence intensity of EGFP (measured in the EGFP transduced areas of GCL and IPL, and normalised to length of the analysed area of the retina) was defined as the sum of fluorescence intensity of each pixel of the analysed area and measured in cellSens; 4 measurements/retina were taken; n = 4. Automated colocalisation of EGFP and RBPMS immunolabels and automated counting of EGFP positive cells in the INL were carried out using the Count and Measure module in cellSens and were normalised to the length of the measured area of the retina. All cells in two stitched frames/retina were counted; n = 4. Amacrine cells (RBPMS negative/PAX6 positive) that expressed EGFP were counted manually; n = 4. Automated colocalisation of BRN3A (RGCs) and EGFP labels in wholemounts were also carried out using the Count and Measure module in cellSens. Six representative areas of each wholemount were analysed and the average of these measurements calculated (n = 5). Images taken with different filter sets were post-processed in Photoshop CS6 and 2020 (Adobe) for the figures; the same settings/operations were applied for all images both in cellSens and Photoshop.

### Statistical analysis

Student’s *t*-test and ANOVA (with Tukey’s multiple comparisons post-hoc test) were performed using Prism 8 (GraphPad) and p < 0.05 was considered statistically significant.

### Ethical statement

Ethical approval for this work was provided by the Irish Medicines Board (now Health Products Regulatory Authority) pursuant to the European Union (Protection of Animals Used for Scientific Purposes) Regulations 2012 (SI 543/2012). All work was submitted to and approved by the Animal Research Ethics Committee (AREC) of Trinity College Dublin.

## Supplementary information

Supplementary Information.
