# Supplementary material for: Novel 199 base pair NEFH promoter drives expression in retinal ganglion cells
Source: Sci Rep. 2020 Oct 5;10:16515. doi: 10.1038/s41598-020-73257-z (PMC7536420; doi:10.1038/s41598-020-73257-z)
Supplement: Supplementary file 1 — Supplementary Information. [file 41598_2020_73257_MOESM1_ESM.docx]

Novel 199 base pair *NEFH* promoter drives expression in retinal ganglion cells

Sophia Millington-Ward^1*^ ^ƚ^, Naomi Chadderton^1^ ^ƚ^, Megan Berkeley^1^, Laura K Finnegan^1^, Killian S Hanlon^1^, Matthew Carrigan^1^, Peter Humphries^1^, Paul F Kenna^1,2^, Arpad Palfi^1 ł^, G Jane Farrar^1 ł^

Submission ID 259409db-bdf3-4021-96e0-62cbcb7c5501

Supplemental table 1

| *NEFH-P* | CAGAATACATCTTGGAGCCCCCTTTTTACCCCAAACCCCCATTCCTCCTTGCTGTCAGCTGCTTGTGAGCCTTCTCACATCCAGAGAATGTATCAGCATTGTGCAGACTGAAAAGACCCAGAGGAACAAGGCTCCAATGGCAAAATTCCAAGTAGAATGACAAATAAATGGGGAGCCATCTGAGAGCAAGGGAGTCCTGCCCAACACCCGCCCCATGCCTTTCTCAGGGACCTCAGACCAGCCACTCACCTCCATCCTCCCAGCACCACCTGCAACCAGCCCCTTGCCCTCTGCAAACTGGAGCACGACTGGATCTTTAGATGGGGGAAAAATGCTTCATCATGTTCTGCTGCTTCATGCAAAACCAGAAACTCCCTCCCCCTCTTCCCTCCTCCCAGCGCACTCTCCTTCCAGTAAAAAGTGGTTAAAGGGACAGCGCCATCAATTTCCCAGCTCTGAGGGTCTGCTTAGAACTAGGGGGCTGGAAGGAGACAGAGGGCAAAGAGAAAGGAACTGGCAGAGGTCTTTCCTGGGGGATATGTCTGTTCTGTCCTGGGGATCCTGGAGCAGGAAAACCCGCGTAAAGTAGGGGTGTAGTGGGTGTTGAGATAACTGCCTGGGGGAGGTTCAGAGTGGAAGTACGAGTCTACAAACTCTCAAGGGCGTCTCAGGGCTCCCAGCATCCCCAGGGGTCCTTTCGCAGGGGTCCCTAAGCAGGAGGGGAACAGCCCAGAAAACACGGAACTGGACCCCCGACAGGAAGTCCAGGGAGGGGTCCCTGGCTCACTATGTGACCCTGCTGGATCACTTGCCTCCCCTCTCGGGTCCCCTCAGCACAGTGTCCCTCCCTTCCTTCCCCTAAAGTAAAAGCAGAGGGTTAATCTCTTTCCCCGCCCCACGCCCAACAAAGAGCAGGCCCTGTCCCCGGTGCTGAAGCGCCAGCCGCAGCACCACCCCCACTCCCACAGCATAAAACATGAGCCAAAACCAATAAAGAGCCAAATGTCACAGCCGTTGCAGGGCCCCCTAAATCCTGGGGACCCCTTCTTCTACCTGACATCCTATTGGGGTGAGGGACTTTGGTACTCAGAAAGCATCTCATCACTTCCCTGTAAGAGAGAAGGGATGCCGACTCAGGCGCCTGCTTGTCTGTTACAGGAGTGGGGGAAGAGAGGACAAGTTGAGGCTGAGAAGATGGGGAGGGGGAGGGAGAAAAGAGGACTTCCTAGTGTTGACAGAACGGCAAGATGTGGGTTCCCCATCCCCAGTTCAGCCAGAGACCCCTCAAAGTGGAACTTCCTGGGGCAGTCGGGGGTCAGGAGTTGGAGCTTGTCTCTGGGGCAAGACCCCTTCGTTGTACAGATGGAAAAACAAGGGTGGGAGGACACAGCTTGTCCAAGGTCATTCGACCAGCAAACTGCCTAGCTGACCCCAGTGTGCAGAAGCTGGCTCGGGTGACACCCATCATTTCCCCCCACCCCACACAGGGGCCAGCTCTCTCAACTTCATGCCCAAGCCCTCCTACGGTACCCCCACTGTAGGTTCTCTGCCCCTCAAACTCAGCCCAGCTTTCTCCTGCCTGTTCAGGGGACCTTCTGCCCGCTTCGCTGAGGGTCCGTCCCCTTTACTGGGGCTGGCAGCAGGGTCTCCCATCTCCTCTCTCGGGGGCCACTGCAGACTTTTTAGAGAACGCCTTGCCTCCCCCCAACCCCACCCATCCGGGGTTCCCTCTCTCCATCCTCTGCAGTGTCTCCCATACCCCCATTCAGGGTAGCCTTGCTATTCTCCCCAACTCCAGGTCCCCCTTCATCTATTCCGGGGCTGGCCGCGGAGTTTCCTGAGCGCTCTCCAAGTGGGTCCTCTAGATGTTAGGAGAACACTGTACCTCCCCCGGTCAGGGGTCTCCTGTCTCCGTTCTATGGAGCGTCCATGCTCCCATTCAGGACTGCCTTGCTCCCTCCTCTGTTCCGGGGCTGGCTGCACAGTCTCTGCACCCCCTATCCTGAAAGCCTCTCTTAACTATTTGGAAAGCCTCGTGTCCTGTCTCATACAGGGATCCCCTCATCCTAATGACTGCAATCTTCCATTGCTCCATCCCGAGGGCATCCTGCCCCTATTCCCATCAGGTTTCTCCTTGTCCTCTCCCTGTTTCAAGTCCCCTTTCTTATTCCGAACACACTCGCAGGCTCTTCCGACGCGCACCCGGGGGTCCTCACTGGCCCACTCCGGGAGTCCTCTGCCCGCTTCCCCGACCTCGAGGGTCTCCTCTGACGCAGCGTCGATTCCCCTTCCCTCCTCGGTCCCCTGCCCCGCCCCTCTCACTGCGGCGGAGCCGGTCGGCCGGGGGGCCGCAGGGGAGGAGGCGGAGAGGGCGGGGCCCTCCTCCCCACCCTCTCACTGCCAAGGGGTTGGACCCGGCCGCGGCGGCTATAAAAGGGCCGGCGCCCTGGTGCTGCCGCAGTGCCTCCCGCCCCGTCCCGGCCTCGCGCACCTGCTCAGGC |
| --- | --- |
| *hA-P* | CCCTGCCCCGCCCCTCTCACTGCGGCGGAGCCGGTCGGCCGGGGGGCCGCAGGGGAGGAGGCGGAGAGGGCGGGGCCCTCCTCCCCACCCTCTCACTGCCAAGGGGTTGGACCCGGCCGCGGCGGCTATAAAAGGGCCGGCGCCCTGGTGCTGCCGCAGTGCCTCCCGCCCCGTCCCGGCCTCGCGCACCTGCTCAGGC |

**Supplemental Table 1. *NEFH-P* and *hA-P* sequences.** Conserved regions *hF* and *hA* are highlighted in green in the *NEFH-P* sequence.
